# Correlation among Lens Opacities Classification System III grading, the 25-item National Eye Institute Visual Functioning Questionnaire, and Visual Function Index-14 for age-related cataract assessment

**DOI:** 10.1007/s10792-020-01353-0

**Published:** 2020-04-05

**Authors:** Yu Wan, Yinhao Wang, Liming Zhao, Min Sun, Li An, Yang Yang, Aimin Jiang, Yanhui Xu, Zhimin Chen, Xuemin Li

**Affiliations:** 1 Peking University Third Hospital, Beijing, China; 2 Beijing Fengtai Hospital, Beijing, China; 3 Huabei Petroleum General Hospital, Renqiu, China; 4 Datong Aier Eye Hospital, Datong, China; 5 The Hospital of Shunyi District Beijing, Beijing, China; 6 Hebei Eye Hospital, Xingtai, China

**Keywords:** Age-related cataract, Cataract types, Subjective visual function, VF-14, NEI-VFQ-25

## Abstract

**Purpose:**

To evaluate the relationship between cataract types and subjective visual function among patients with age-related cataract.

**Methods:**

This was a prospective, multicenter, 831 Chinese patient-based, cross-sectional study. Patients were administered the Visual Function Index-14 (VF-14) and the 25-item National Eye Institute Visual Functioning Questionnaire (NEI-VFQ-25) to evaluate their subjective visual function. Lens Opacities Classification System III (LOCS III) was used to evaluate the type of cataract. Relationships among these parameters were analyzed.

**Results:**

LOCS III cortical (C) and posterior subcapsular scores are negatively associated with VF-14 (*r* = − 0.188, *P *< 0.01; *r* = − 0.146, *P *< 0.01) and total score of NEI-VFQ-25 (*r* = − 0.223, *P *< 0.01; *r* = − 0.160, *P *< 0.01), respectively; LOCS III nuclear opalescence (NO) score is positively associated with VF-14 (*r* = 0.087, *P *< 0.05) and total score of NEI-VFQ-25 (*r* = 0.097, *P *< 0.05). In multiple linear regression, a decrease in the LOCS III C score is a significant predictor for improvement of the total score of NEI-VFQ-25 (*β* = − 1.286, *P *< 0.05). In contrast, an increase in LOCS III NO score is a significant predictor for improvement of VF-14 (*β* = 3.826, *P *< 0.01) and total score of NEI-VFQ-25 (*β* = 4.618, *P *< 0.01). Patients with LOCS III C score ≤ 2 have higher VF-14 (49.38 versus 43.74, *P* < 0.01), total (80.73 versus 71.58, *P* < 0.01) and subscale scores of NEI-VFQ-25 than patients with LOCS III C score > 2.

**Conclusion:**

Cortical cataract has adverse effects on subjective visual function, while mild-to-moderate nuclear cataract has positive effects. Furthermore, “LOCS III C score > 2” can be a potential cutoff as a reference for cataract surgery without self-assessing questionnaires.

**Electronic supplementary material:**

The online version of this article (10.1007/s10792-020-01353-0) contains supplementary material, which is available to authorized users.

## Introduction

Cataract is the primary cause of blindness, which can significantly influence people’s quality of life. Age-related cataract is the most common type of cataract that injuries the visual function and quality of life for the elderly [1, 2]. Cataract surgery can significantly improve vision, visual functioning, and the quality of life of patients [3–7]. Therefore, it is essential to determine the surgical timing for cataract. In order to comprehensively assess the status of patients with cataract, objective and subjective measures were performed and studied [6, 8–10].

The Lens Opacities Classification System III (LOCS III) is one of the most common subjective tools to grade the opacities of the lens at the slit lamp [11, 12]. The Visual Function Index-14 (VF-14) and the 25-item National Eye Institute Visual Functioning Questionnaire (NEI-VFQ-25) are patient-perspective questionnaires that are proven to be reliable and valid [13–19]. These two questionnaires are used as a supplement for existing measurements of cataract [9, 10, 20, 21], such as visual acuity and contrast sensitivity. Several pieces of research studied the relationship between the LOCS III score and the score of vision-specific self-assessing questionnaires [9, 10]. However, this relationship was inconsistent [9, 10].

In the present study, we aim to analyze the relationships among visual acuity, LOCS III scores, VF-14, and NEI-VFQ-25 scores in the Chinese population. We hope to find a potential cutoff of LOCS III scores as a reference for cataract surgery.

## Subjects and methods

### Subjects

This study is prospective, multicenter, Chinese population-based, and cross-sectional. A total of 831 eyes among 831 patients with bilateral age-related cataract from five different centers were enrolled between March and June 2019 in our study. Patients with glaucoma, fundus diseases, amblyopia, history of ocular surgery, and cognitive disorder were excluded in our study. This research was approved by Peking University Third Hospital Medical Science Research Ethics Committee, which was adhered to the tenets of the Declaration of Helsinki. Informed consent was obtained from all the participants.

### Clinical characteristics

Uncorrected distance visual acuity (UDVA) of the operative eye (op-eye) and the non-op eye was recorded. The operative eye is defined as the eye with worse vision or more significant influence on life planned to received cataract surgery. UDVA of the op-eye was categorized into four categories: normal vision (< 0.3 LogMAR), mild vision impairment (0.3 to < 0.5 LogMAR), moderate vision impairment (0.5 to < 1.0 LogMAR), and severe vision impairment and blindness (≥ 1.0 LogMAR). UDVA of the other eye was recorded as well. The opacity of each op-eye was assessed using LOCS III, including nuclear opalescence (NO), cortical (C), and posterior subcapsular (P) scores. A well-trained ophthalmologist graded every eye under slit-lamp examination according to standard color figures of LOCS III grading [22]. We classified the LOCS III C score into two groups, “Group C1” (≤ 2) and “Group C2” (> 2) in our study.

### Self-assessment questionnaires

Two widely used self-assessing questionnaires (VF-14 and NEI-VFQ-25) [19, 23], translated in Chinese, were completed by each subject before surgery to evaluate subjective visual function. The subscales of NEI-VFQ-25 included General Health, General Vision, Ocular Pain, Near Activities, Distance Activities, Social Functioning, Mental Health, Role Difficulties, Dependency, Driving, Color Vision, and Peripheral Vision. A well-trained ophthalmologist would explain the questions on the questionnaires to subjects who cannot figure out or understand the questionnaire by himself/herself. The subscale scores and total scores were calculated according to the scoring algorithm. Systemic diseases influencing the vision, including hypertension and diabetes, were recorded.

### Statistical analysis

IBM SPSS Statistics for Windows (version 20.0. Armonk, NY: IBM Corp) and GraphPad Prism 5 for Windows (version 5.01. GraphPad Software, Inc) were used to analyze the data and draw graphs. Categorical variables were shown as percentage, rank variables, and continuous variables were shown as mean ± SD or median. One sample Kolmogorov–Smirnov test was used to check the normality of each variable. None of the variables were normally distributed. Mann–Whitney *U* test was used to compare the difference between two independent samples. Association among different variables was explored by the Spearman coefficient test. The coefficient *r* < 0.4 indicates a weak correlation; ≥ 0.4–0.7 indicates a moderate correlation; ≥ 0.7 indicates a strong correlation. Multiple linear regression analysis was used to adjust confounding factors. Baseline variables that were considered clinically relevant or that showed a univariate relationship with outcome entered the multiple linear regression model. Finally, nine confounding factors were analyzed for VF-14 and NEI-VFQ-25. According to partial plots and scatter plots of studentized residuals with unstandardized predicted values, a linear relationship exists between independent variables and dependent variables. Durbin-Watson values are 1.567, 1.254 for VF-14, and NEI-VFQ-25, respectively, which means that observations are independent of each other. It is validated that the data is equivariant through a scatter plot between the studentized residuals and the unstandardized predicted values. There is no multicollinearity since tolerance is over 0.1. Through the outlier test, a total of five VF-14 outliers, and six NEI-VFQ-25 outliers were deleted (outlier standard: studentized deleted residuals > 3 times of standard deviation for observations, leverage values > 0.2 or Cook’s distance values > 1). The regression models are statistically significant, with adjusted *R*^2^ = 0.226 and 0.353 for VF-14 and NEI-VFQ-25, respectively. The level of significance is *P* < 0.05.

## Results

**Fig. 1:**
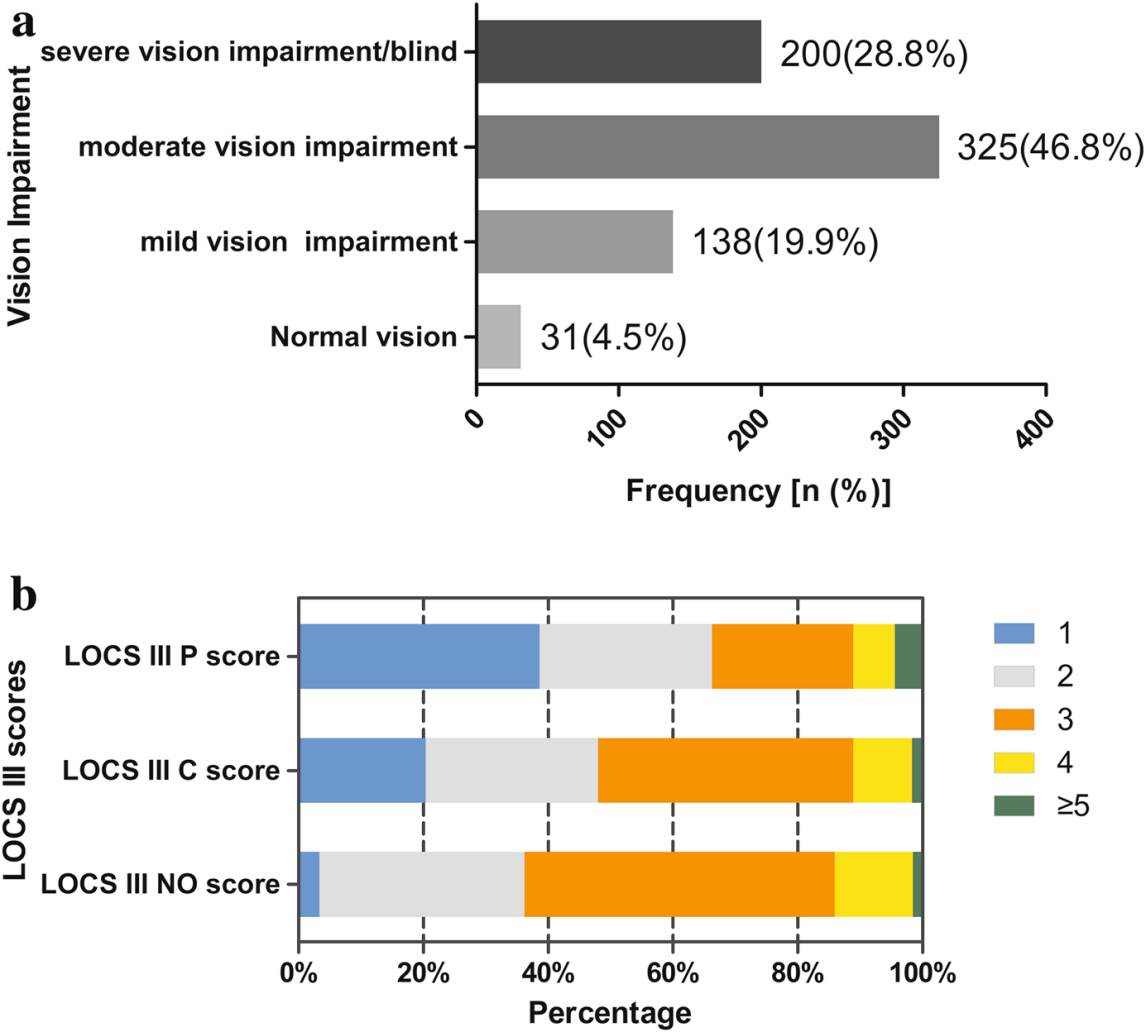
Bar graph showing the distribution of vision impairment, LOCS III NO, C, and P scores among patients with age-related cataract. **a** The distribution of vision impairment. **b** The distribution of LOCS III NO, C, and P scores. *LOCS* Lens Opacities Classification System, *NO* nuclear opalescence, *C* cortical, *P* posterior subcapsular

A total of 831 eyes from 831 patients were enrolled, and there were 137 subjects dropped out for incomplete clinical data or abnormal values. Overall, there were 694 subjects included with a drop-out rate of 16.5%. The demographic and clinical characteristics of subjects are presented in Table 1. UDVAs of the operative eyes range from 0 to 2.0 (LogMAR), and vision impairment is shown in Fig. 1a. Mean LOCS III scores were 2.76 ± 0.78, 2.45 ± 0.97, and 2.11 ± 1.13 for NO, C, and *P*, respectively. Figure 1b shows the distribution of various LOCS III scores. There were 333 eyes (48.0%) in Group C1 and 361 eyes (52.0%) in Group C2. The VF-14 score and the total or subscale scores of NEI-VFQ-25 are presented in Table 1.

**Table 1:** Demographic and clinical characteristics of subjects, VF-14 scores and NEF-VFQ-25 scores (*n* = 694)

Variables	Mean (SD)	Median
Age (year)	70.23 (9.93)	71.00
*UDVA (LogMAR)*		
Op-eye	0.76 (0.39)	0.70
Non-op-eye	0.46 (0.35)	0.40
LOCS III NO score	2.76 (0.78)	3.00
LOCS III C score	2.45 (0.97)	3.00
LOCS III P score	2.11 (1.13)	2.00
VF-14	46.67 (15.02)	45.83
*NEF*-*VFQ*-*25*		
General health	39.66 (19.79)	50.00
General vision	46.31 (16.95)	40.00
Ocular pain	84.78 (20.86)	100.00
Near activities	68.36 (24.66)	75.00
Distance activities	77.61 (23.62)	83.33
Social functioning	86.62 (22.31)	100.00
Mental health	78.87 (24.82)	93.75
Role difficulties	71.52 (29.31)	75.00
Dependency	81.15 (26.98)	100.00
Driving (*n* = 224)	73.23 (35.08)	91.67
Color vision	86.78 (25.11)	100.00
Peripheral vision	79.57 (25.62)	100.00
Total score	76.16 (18.87)	82.21

Number (%)	
Gender (male/female)	273 (39.3)/421 (60.7)
Hypertension (no/yes)	406 (58.5)/288 (41.5)
Diabetes (no/yes)	534 (76.9)/160 (23.1)

*SD* standard deviation, *UDVA* uncorrected distance visual acuity, *Op*-*eye* operative eye, *NO* nuclear opalescence, *C* cortical, *P* posterior subcapsular, *LOCS III* Lens Opacities Classification System III, *VF*-*14* Visual Function Index-14, *NEI*-*VFQ*-*25* 25-item National Eye Institute Visual Functioning Questionnaire

**Fig. 2:**
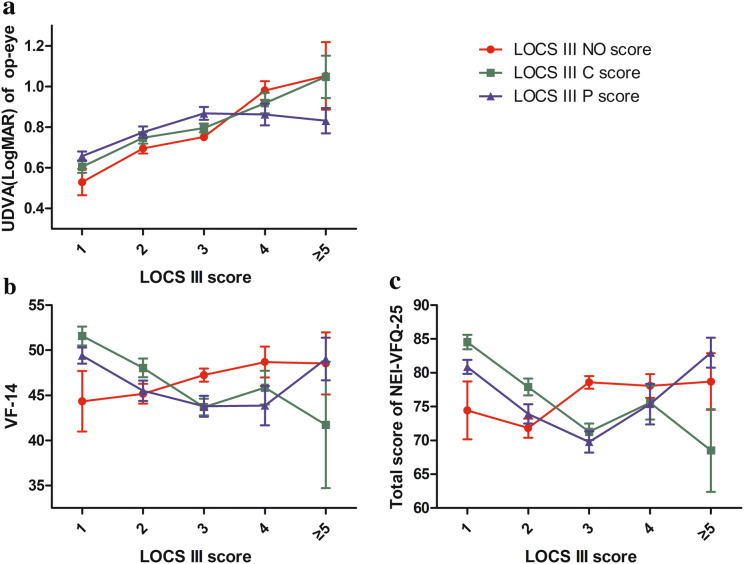
Line graph showing the correlation among UDVA, the VF-14 score, the total score of NEI-VFQ-25, and LOCS III scores. **a** UDVA of the op-eye changing with LOCS III scores. **b** The VF-14 score changing with LOCS III scores. **c** The total score of NEI-VFQ-25 changing with LOCS III score. *UDVA* uncorrected distance visual acuity, *op-eye* operative eye, *VF-14* the Visual Function Index-14, *NEI-VFQ-25* the 25-item National Eye Institute Visual Functioning Questionnaire, *LOCS* Lens Opacities Classification System, *NO* nuclear opalescence, *C* cortical, *P* posterior subcapsular. Error bars indicate SEM

Spearman correlation test was applied among age, LOCS III NO, C, P scores, UDVA, and each scale score (Table 2). LOCS III C, P scores are negatively correlated with the VF-14 score, total and most subscale scores of NEI-VFQ-25, whereas LOCS III NO score is positively correlated with the VF-14 score, total and most subscale scores of NEI-VFQ-25. UDVA of either eye is negatively correlated with the VF-14 score and the total score of NEI-VFQ-25 (Table 2). A line graph was plotted to illustrate the association among LOCS III scores, UDVA, the VF-14 score, and the total score of NEI-VFQ-25 (Fig. 2). With the increase in the LOCS III NO score, UDVA decreases, while the VF-14 score and the total score of NEI-VFQ-25 tend to increase. With the increase in the LOCS III C score, UDVA, the VF-14 score, and the total score of NEI-VFQ-25 decrease. For the LOCS III P score, a U-shaped tendency is presented, and “3” seems to be a turning point.

**Table 2:** Correlation between characteristics and self-reported questionnaire scores (*n* = 694)

Scale scores	Age (year)	LOCS III scores			UDVA (LogMAR)	
		NO	C	*P*	Op-eye	Non-op-eye
VF-14	− 0.069	0.087*	− 0.188**	− 0.146**	− 0.344**	− 0.317**
General health	− 0.188**	0.019	− 0.066	0.013	− 0.099**	− 0.230**
General vision	− 0.094*	− 0.126**	− 0.050	− 0.029	− 0.221**	− 0.328**
Ocular pain	0.071	0.169**	− 0.184**	− 0.144**	− 0.212**	− 0.239**
Near activities	− 0.120**	0.043	− 0.152**	− 0.117**	− 0.275**	− 0.379**
Distance activities	− 0.083*	0.117**	− 0.197**	− 0.150**	− 0.369**	− 0.494**
Social functioning	− 0.021	0.138**	− 0.218**	− 0.183**	− 0.322**	− 0.347**
Mental health	− 0.037	0.140**	− 0.194**	− 0.143**	− 0.329**	− 0.373**
Role difficulties	− 0.019	0.082*	− 0.224**	− 0.094*	− 0.300**	− 0.399**
Dependency	− 0.033	0.141**	− 0.177**	− 0.161**	− 0.316**	− 0.369**
Driving	− 0.012	0.020	− 0.265**	0.007	− 0.229**	− 0.333**
Color vision	− 0.012	0.133**	− 0.208**	− 0.171**	− 0.296**	− 0.301**
Peripheral vision	− 0.044	0.134**	− 0.184**	− 0.182**	− 0.342**	− 0.401**
Total score of NEI-VFQ-25	− 0.050	0.097*	− 0.223**	− 0.160**	− 0.376**	− 0.497**

*P < 0.05; **P < 0.01

*UDVA* uncorrected distance visual acuity, *Op*-*eye* operative eye, *NO* nuclear opalescence, *C* cortical, *P* posterior subcapsular, *LOCS III* Lens Opacities Classification System III, *VF*-*14* Visual Function Index-14, *NEI*-*VFQ*-*25* 25-item National Eye Institute Visual Functioning Questionnaire

**Fig. 3:**
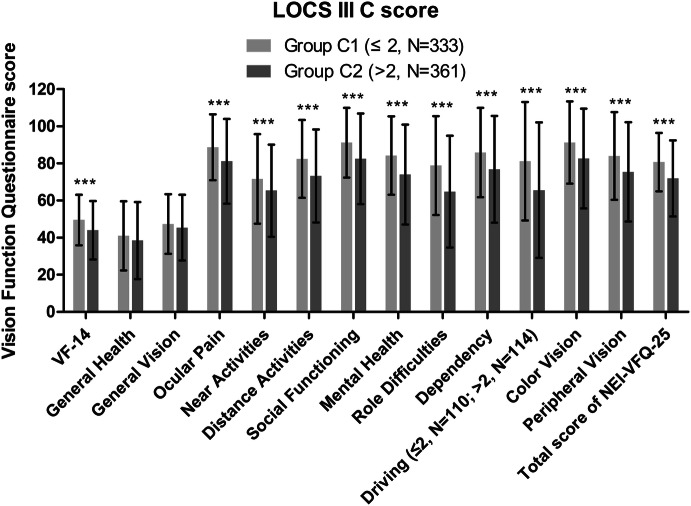
Bar graph showing the difference of UDVA, the VF-14 score, the total and subscale scores of NEI-VFQ-25 score between Group C1 (LOCS III C score ≤ 2) and Group C2 (LOCS III C score > 2). *UDVA* uncorrected distance visual acuity; *VF-14* the Visual Function Index-14; *NEI-VFQ-25* the 25-item National Eye Institute Visual Functioning Questionnaire. ***P < 0.001 analysis of variance. Error bars indicate SD

Mann–Whitney *U* test shows that male has a higher total score of NEI-VFQ-25 compared with female (78.93 ± 17.94 versus 74.36 ± 19.25, *P* < 0.01); patients with hypertension have a higher total score of NEI-VFQ-25 (80.98 ± 15.82 versus 72.74 ± 20.09, *P* < 0.01) and a higher VF-14 score (48.58 ± 13.71 versus 45.32 ± 15.76, *P* < 0.01); patients with diabetes have a higher total score of NEI-VFQ-25 (80.49 ± 15.73 versus 74.86 ± 19.54, *P* < 0.01). Patients in Group C1 have a higher VF-14 score, higher total and subscale scores of NEI-VFQ-25 than in Group C2 (Supplementary Table, Fig. 3).

Multiple linear regression analysis for VF-14 and NEI-VFQ-25 is presented in Table 3.

**Table 3:** Multiple linear regression model for VF-14, total score of NEI-VFQ-25

	VF-14			Total score of NEI-VFQ-25		
	*β*	SE	SC	*β*	SE	SC
(Constant)	66.056	4.255	–	96.829	4.828	–
Age	− 0.177	0.053	− 0.120**	− 0.094	0.061	− 0.052
Gender	− 1.432	1.013	− 0.048	− 4.027	1.151	− 0.108**
UDVA of op-eye	− 11.261	1.474	− 0.294**	− 13.176	1.662	− 0.280**
UDVA of non-op-eye	− 9.731	1.508	− 0.232**	− 17.955	1.712	− 0.345**
LOCS III NO score	3.826	0.712	0.203**	4.618	0.809	0.198**
LOCS III C score	− 0.878	0.533	− 0.058	− 1.286	0.604	− 0.069*
LOCS III P score	− 0.399	0.448	− 0.031	− 0.558	0.510	− 0.035
Hypertension	1.369	1.056	0.046	4.999	1.195	0.136**
Diabetes	0.706	1.214	0.020	3.048	1.378	0.071*

*P < 0.05; **P < 0.01

*SE* standardized error, *SC* standardized coefficients, *UDVA* uncorrected distance visual acuity, *Op*-*eye* operative eye, *NO* nuclear opalescence, *C* cortical, *P* posterior subcapsular, *LOCS III* Lens Opacities Classification System III, *VF*-*14* Visual Function Index-14, *NEI*-*VFQ*-*25* 25-item National Eye Institute Visual Functioning Questionnaire

## Discussion

The mean of UDVA (LogMAR) of op-eye in our study is 0.76, which is better than severe vision impairment or blindness. Among these patients undergoing cataract surgery, only 28.8% of them had severe vision impairment or blindness. Most of them are patients with moderate vision impairment. We also noticed that even patients with no apparent vision impairment were going to receive surgery. Patients with cataract used to receive surgery when they had to, but now they determine to receive surgery when their vision cannot meet their needs in life, such as the demand for driving. These phenomena indicate that cataract surgery is gradually transforming from an “unblinding surgery” to a “refractive surgery” for better vision-specific quality of life.

Some studies found a stronger association between subjective and objective visual function among the better eyes, rather than the worse eyes or operative eyes [8, 9, 19, 24]. Our data showed a significant association between UDVA of the op-eye or non-op-eye and self-assessing questionnaires, even after adjusting confounding factors. In the multiple regression model, UDVA of op-eye has a more remarkable effect on the VF-14 score, while UDVA of non-op-eye has a more prominent effect on the total score of NEI-VFQ-25. Such paradox potentially revealed that these two questionnaires reflect the vision function of different eyes. Besides, we analyzed the association of the VF-14 score and the total score of NEI-VFQ-25, in which they were moderately correlated with each other. Therefore, we probably chose VF-14 to evaluate the influence of the op-eye on subjective vision function. Otherwise, NEI-VFQ-25 would be a better choice for the non-op-eye. The correlation between UDVA and scores of self-assessing questionnaires is not that strong, which is consistent with previous studies [8, 14, 25]. Therefore, such self-assessing questionnaires like VF-14 or NEI-VFQ-25 are much necessary to assist ophthalmologists in evaluating the patients with cataract. However, either VF-14 or NEI-VFQ-25 is too complicated for some patients to understand and for doctors to operate or calculate, and this complexity makes these questionnaires hard for a widespread use. Therefore, it is urgently needed to develop a much simpler scale to screen or evaluate patients.

Chew et al. [26] showed that all types of cataract negatively affected the VF-14 score. Except for nuclear cataract, the results of cortical cataract and posterior subcapsular cataract in another study were similar [10]. These phenomena are consistent with our findings. In the multiple regression model, LOCS III NO score and C score are significantly associated with subjective visual function except for the LOCS III P score. We notice that, when LOCS III NO score < 4, patients with higher LOCS III NO scores tend to have a better subjective visual function, which is against the previous studies [9, 26]. It may not be surprising since a myopic shift caused by nuclear cataract [27] partially corrects the presbyopia, which probably helps improve the subjective visual function. Therefore, for nuclear cataract, it is probably not necessary to receive surgery that radically. Cortical cataract is negatively associated with subjective visual function. To find a cutoff for the LOCS III C score associated with visual function, we divided the data into two groups, Group C1 (LOCS III C score ≤ 2) and Group C2 (LOCS III C score > 2). We found that patients in Group C2 had a significantly worse subjective and objective visual function. As we mentioned above, it is hard to apply such questionnaires widely. This cutoff could reflect the situation of patients’ subjective visual function. Therefore, we recommend patients with higher LOCS III C score or severe cortical change of lens to receive surgery more actively, and “LOCS III C score > 2” should be a potential cutoff as a reference for cataract surgery.

In conclusion, our study suggests that patients with age-related cataract are willing to receive cataract surgery as their visions are not able to satisfy their needs in life at present, even their visual acuities are acceptable. This phenomenon may drive the transformation of the existing medical model of cataract surgery, from an “unblinding surgery” to a “refractive surgery” for better vision-specific quality of life. Nuclear, cortical, and posterior subcapsular cataracts can affect the subjective and objective visual function, respectively. Cortical cataract is negatively associated with subjective visual function, while nuclear cataract has an inverse trend. “LOCS III C score > 2” is probably a potential cutoff as a reference for cataract surgery without self-assessing questionnaires.

## Electronic supplementary material

Below is the link to the electronic supplementary material.

Supplementary material 1 (DOCX 2105 kb)

Supplementary material 2 (DOCX 17 kb)
