# Supplementary material for: Correlation among Lens Opacities Classification System III grading, the 25-item National Eye Institute Visual Functioning Questionnaire, and Visual Function Index-14 for age-related cataract assessment
Source: Int Ophthalmol. 2020 Apr 5;40(7):1831–9. doi: 10.1007/s10792-020-01353-0 (PMC7308262; doi:10.1007/s10792-020-01353-0)

**Supplementary. Questionnaires**

**1. Visual Function Index-14**

**English**

**VF-14 QOL Questionnaire**

**Because of your vision** how much difficulty do you have with the following activities?

Check the box that best describes how much difficulty you have, even with glasses.

| Activity | None | A little | Moderate | Great deal | Unable to do |
| --- | --- | --- | --- | --- | --- |
| 1. Reading small print, such as medicine bottle labels, a telephone book, or food labels | 🗆 | 🗆 | 🗆 | 🗆 | 🗆 |
| 2. Reading a newspaper or a book | 🗆 | 🗆 | 🗆 | 🗆 | 🗆 |
| 3. Reading a large-print book or large-print newspaper or numbers on a telephone | 🗆 | 🗆 | 🗆 | 🗆 | 🗆 |
| 4. Recognizing people when they are close to you | 🗆 | 🗆 | 🗆 | 🗆 | 🗆 |
| 5. Seeing steps, stairs or curbs | 🗆 | 🗆 | 🗆 | 🗆 | 🗆 |
| 6. Reading traffic signs, street signs or store signs | 🗆 | 🗆 | 🗆 | 🗆 | 🗆 |
| 7. Doing the fine handwork like sewing, knitting, crocheting, carpentry | 🗆 | 🗆 | 🗆 | 🗆 | 🗆 |
| 8. Writing checks or filling out forms | 🗆 | 🗆 | 🗆 | 🗆 | 🗆 |
| 9. Playing games such as bingo, dominos, card games, or mahjong | 🗆 | 🗆 | 🗆 | 🗆 | 🗆 |
| 10. Taking part in sports like bowling, handball, tennis, golf | 🗆 | 🗆 | 🗆 | 🗆 | 🗆 |
| 11.Cooking | 🗆 | 🗆 | 🗆 | 🗆 | 🗆 |
| 12. Watching television | 🗆 | 🗆 | 🗆 | 🗆 | 🗆 |
| 13.Driving during the day | 🗆 | 🗆 | 🗆 | 🗆 | 🗆 |
| 14. Driving at night | 🗆 | 🗆 | 🗆 | 🗆 | 🗆 |

**Translated in Chinese**

**视功能指数量表**

**请根据实际情况填写以下内容**

您做以下事情的困难程度：

| 内容 | 极好 | 良好 | 尚可 | 困难 | 无法完成 |
| --- | --- | --- | --- | --- | --- |
| 1、看小字体（如药瓶上的说明书、通讯录、价格标签、银行单据、水费电费单） |  |  |  |  |  |
| 2、读书看报 |  |  |  |  |  |
| 3、看大字体（如电话上的数字按键、挂钟、日历） |  |  |  |  |  |
| 4、认出身旁的人 |  |  |  |  |  |
| 5、看清楼梯、台阶和路缘石 |  |  |  |  |  |
| 6、看清各种标识牌（如交通标志、路标、商店标牌） |  |  |  |  |  |
| 7、做精细活（如编织、缝纫、使用手工工具） |  |  |  |  |  |
| 8、填表或签名 |  |  |  |  |  |
| 9、参加娱乐活动（如麻将、扑克牌、象棋） |  |  |  |  |  |
| 10、参加体育活动（如散步、做操、太极） |  |  |  |  |  |
| 11、做饭 |  |  |  |  |  |
| 12、看电视 |  |  |  |  |  |
| 13、白天驾车 |  |  |  |  |  |
| 14、夜晚驾车 |  |  |  |  |  |

**2.The 25-item National Eye Institute Visual Functioning Questionnaire**

**English**

**
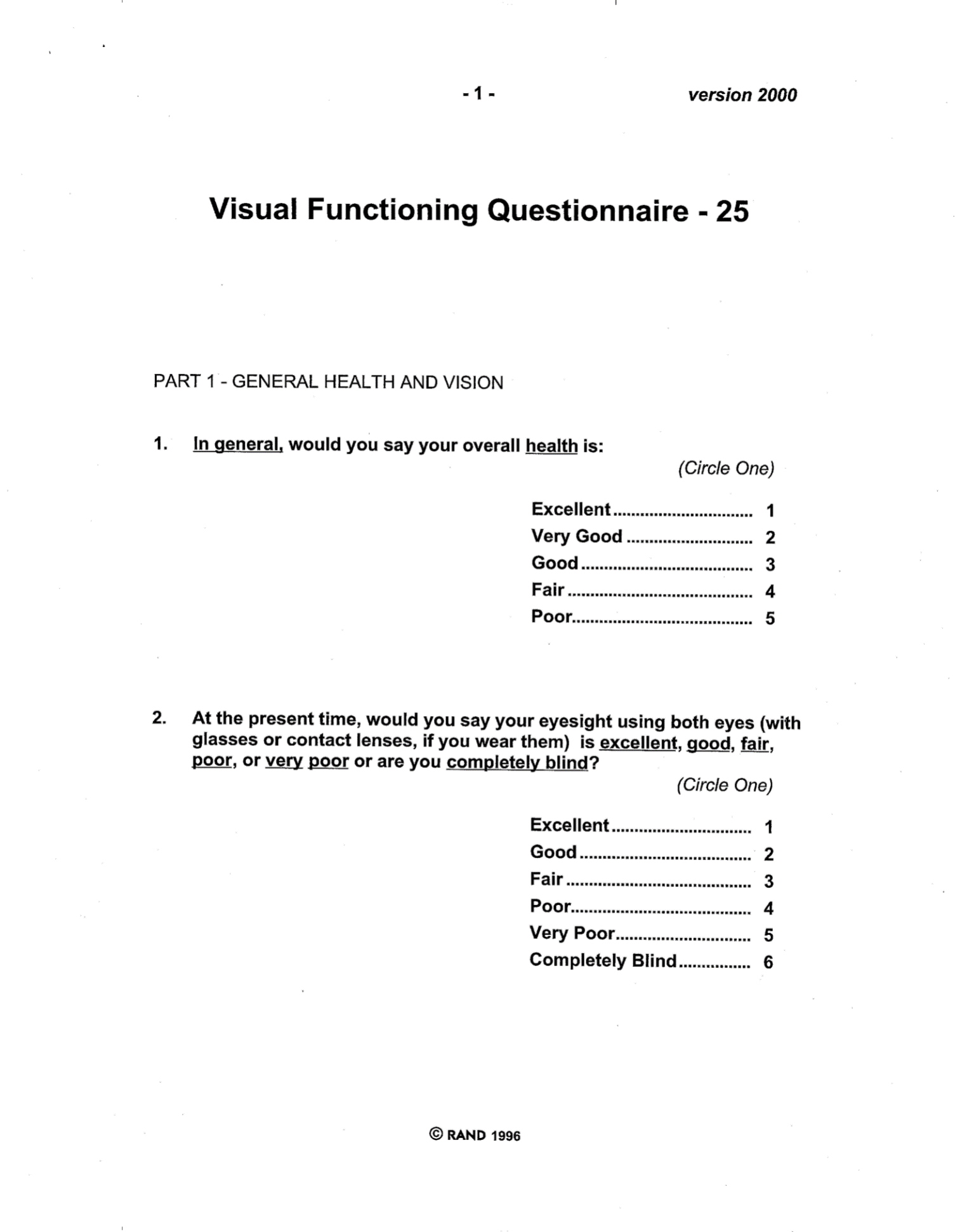
**


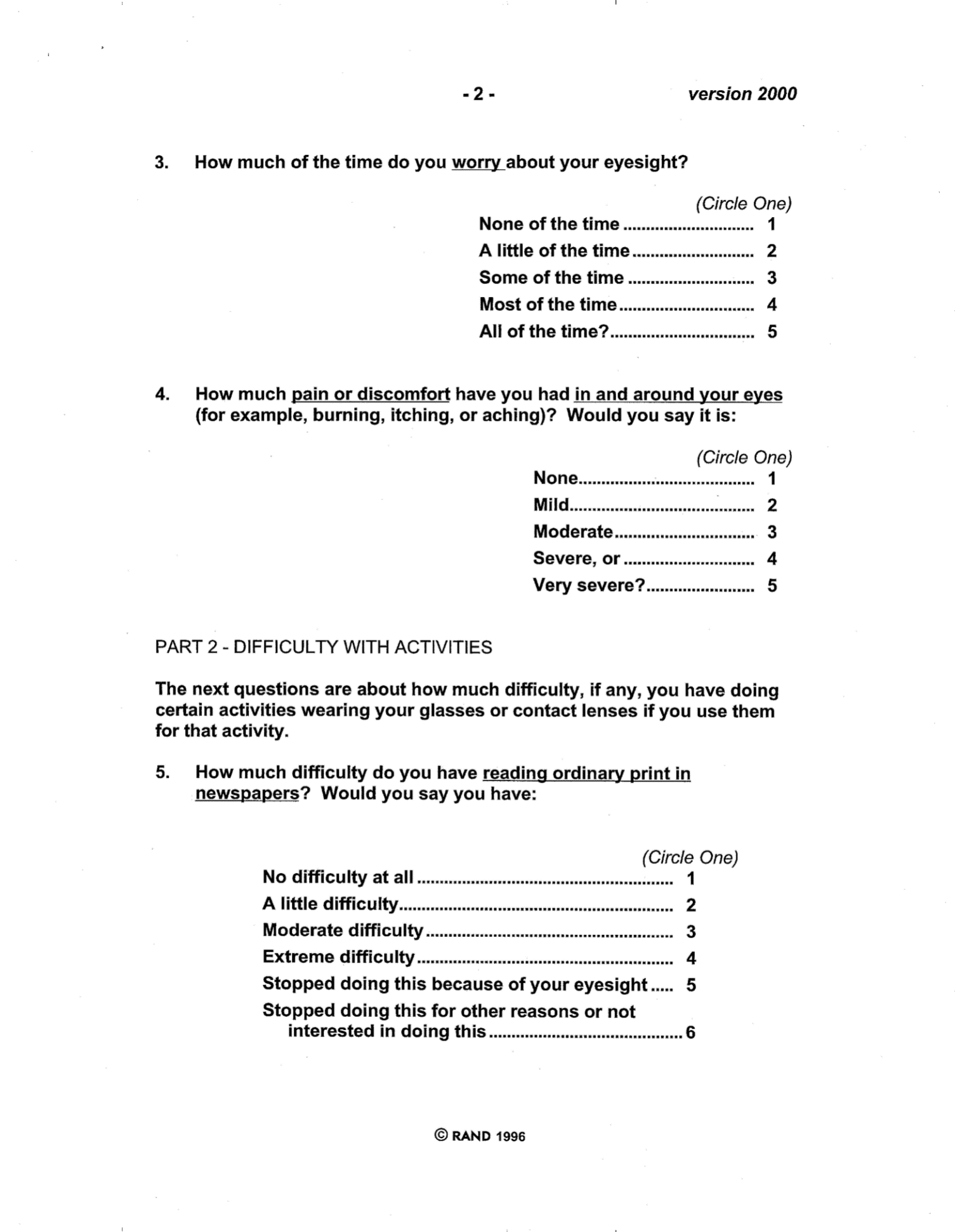


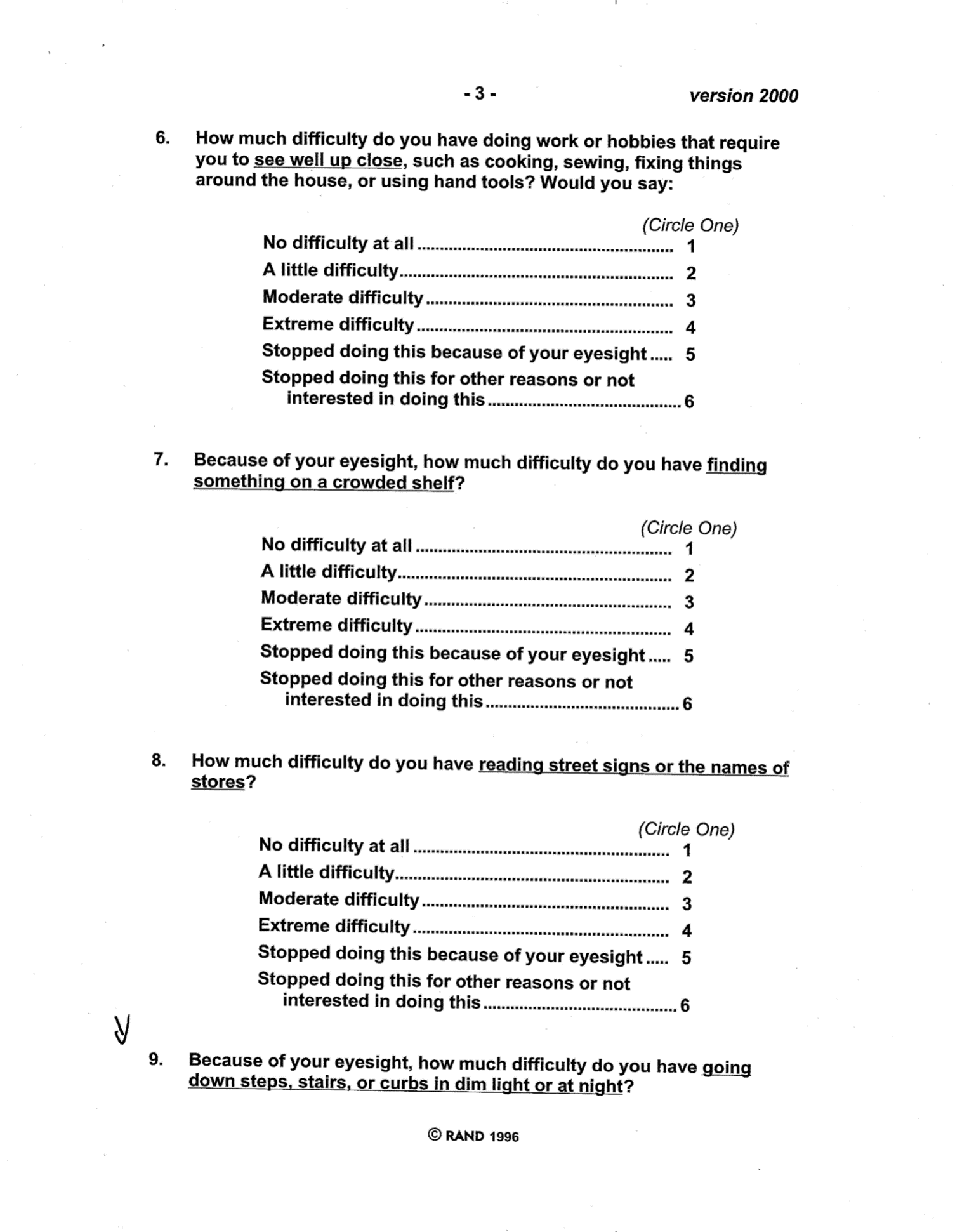


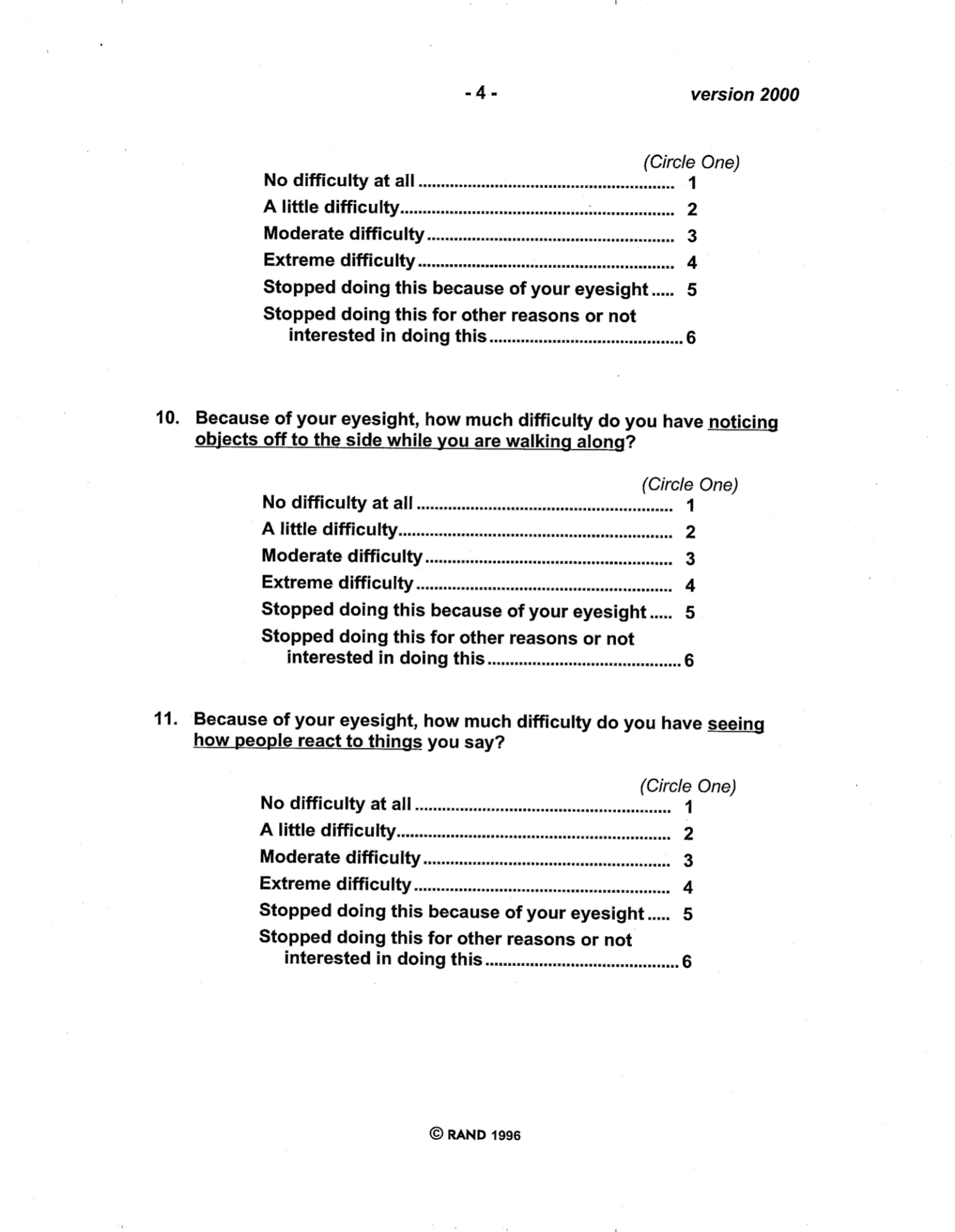


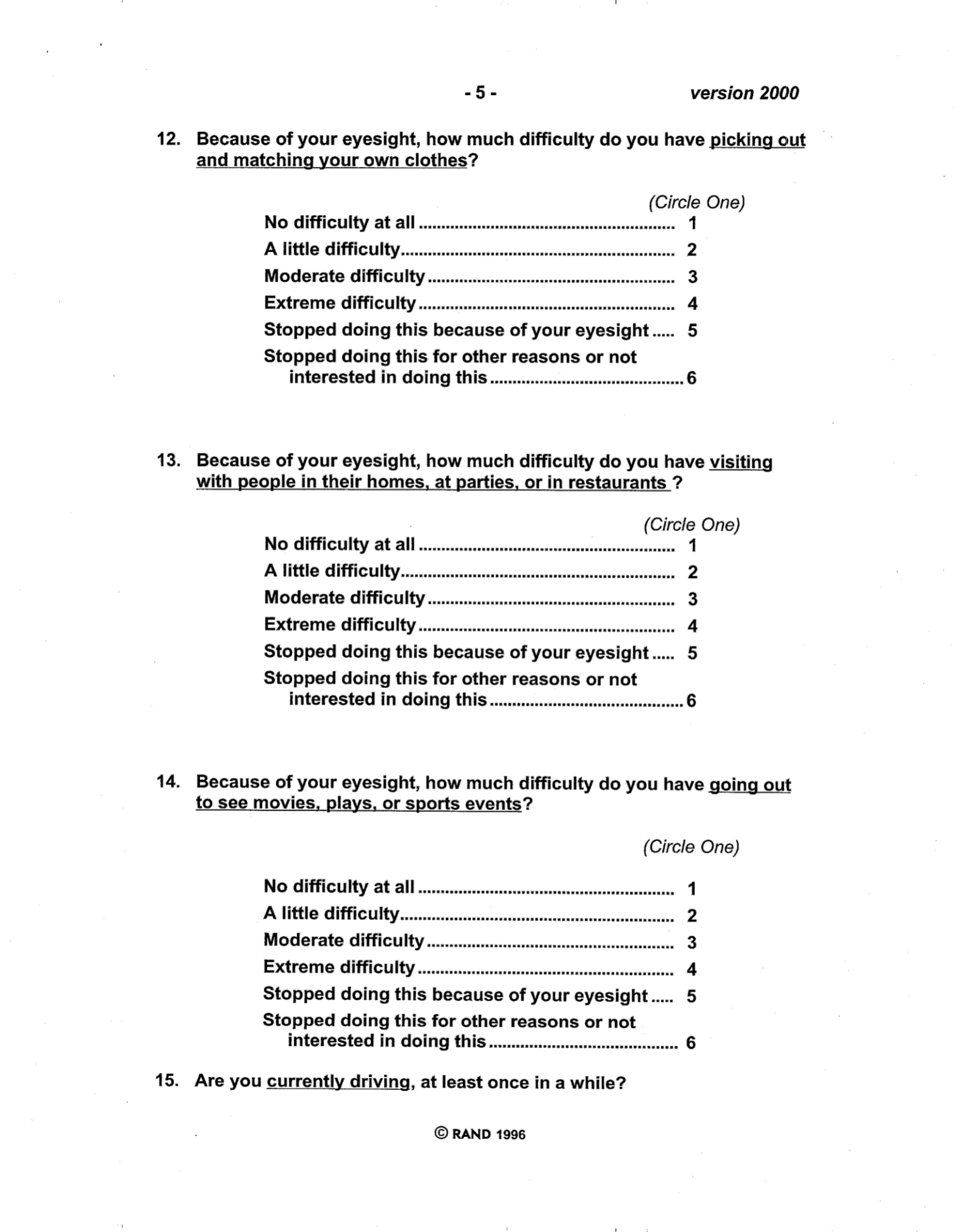


**
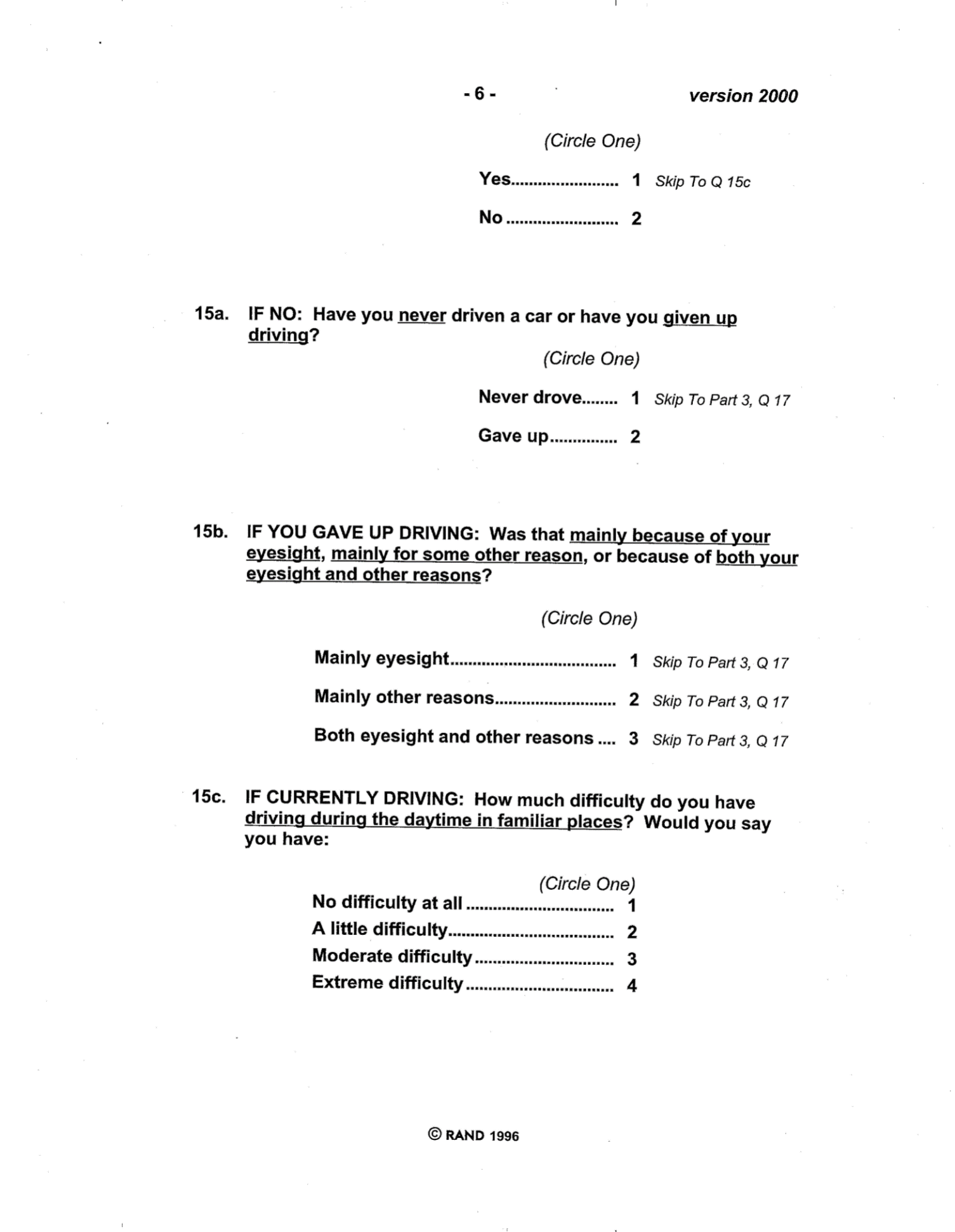
**

**
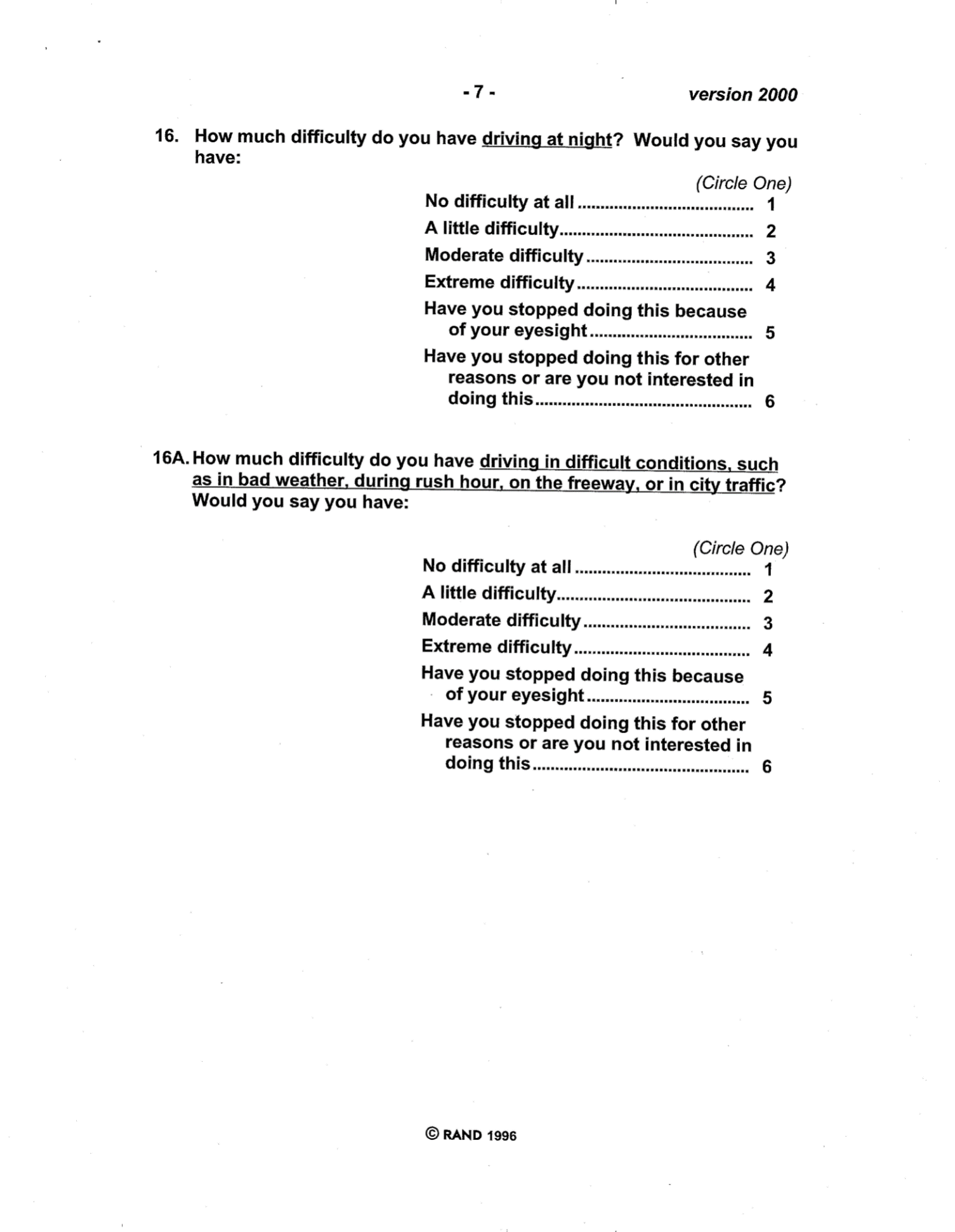
**


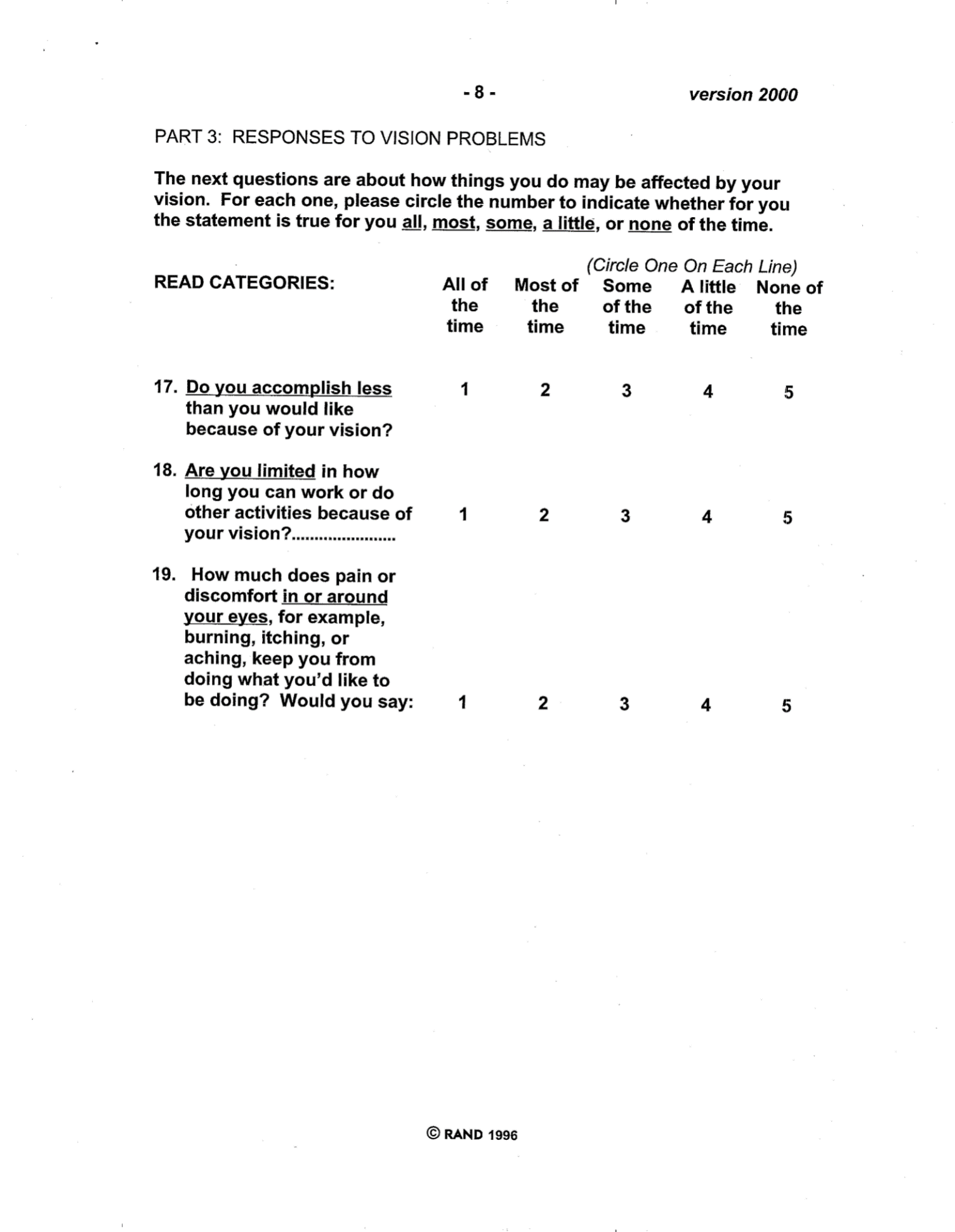


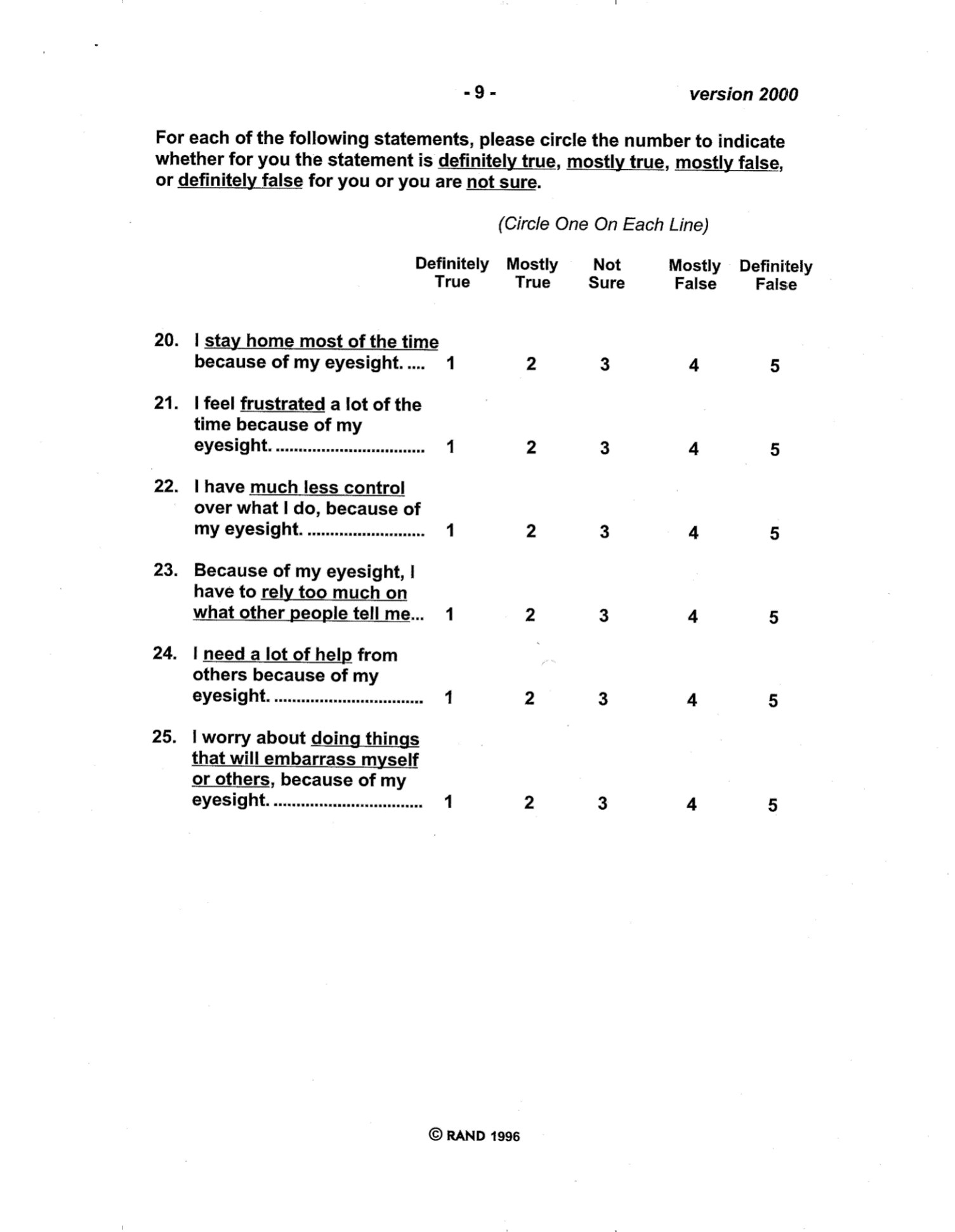


**Translated in Chinese**

**视功能相关的生存质量量表**

第一部分 一般健康及视力情况

1、总体来讲，您自我感觉自己的健康状况？( )

A.极好 B.很好 C.好 D.尚可 E.差

2、目前您的双眼视力(矫正视力)如何？( )

A.极好 B.好 C. 尚可 D. 差 E.极差 F.完全看不到

3、您经常担心自己的视力状况吗？( )

A.从不担心 B.有一点担心 C.有时候担心 D.大多数时间担心 E.一直都担心

4、您的眼睛及眼睛周围有无疼痛或不舒适感(如烧灼感、瘙痒或疼痛等)吗? ( )

A.没有 B.轻微的 C.中等程度的 D.较重的 E.很严重的

第二部分 活动时困难程度

5、您在阅读报纸上普通的字体有多大的困难？( )

A.没有困难 B.有一点困难 C.有困难(中等程度) D.的确很困难

E.由于视力原因已不再阅读 F.由于其它原因或没有兴趣而不阅读报纸

6、当做一些需要看得更清晰的事情时(例如做饭、针线活、在房间周围钉东西或者需要使用工

具)，您有多大困难? ( )

A.没有困难 B.有一点困难 C.有困难(中等程度) D.的确很困难

E.由于视力原因已不再做此事 F.由于其它原因或没有兴趣而不做此事

7、由于视力原因，您在拥挤的货架或书架上寻找东西时有多大困难? ( )

A.没有困难 B.有一点困难 C.有困难(中等程度) D.的确很困难

E.由于视力原因已不再做此事 F.由于其它原因或没有兴趣而不做此事

8、在街道上，您对看清街上的道路标志或商店名称有多大的困难? ( )

A.没有困难 B.有一点困难 C.有困难(中等程度) D.的确很困难

E.由于视力原因已不再做此事 F.由于其它原因或没有兴趣而不做此事

9、由于视力原因，在昏暗的灯光下或晚上，您在下楼梯、台阶时有多大的困难? ( )

A.没有困难 B.有一点困难 C.有困难(中等程度) D.的确很困难

E.由于视力原因已不再做此事 F.由于其它原因或没有兴趣而不做此事

10、由于视力原因，当沿街行走时，您对看清马路上物体旁边的东西有多大困难? ( )

A.没有困难 B.有一点困难 C.有困难(中等程度) D.的确很困难

E.由于视力原因已不再做此事 F.由于其它原因或没有兴趣而不做此事

11、由于视力原因，当与他人交谈时，您在看清对方对您所说事情的反应时有多大困难? ( )

A.没有困难 B.有一点困难 C.有困难(中等程度) D.的确很困难

E.由于视力原因已不再做此事 F.由于其它原因或没有兴趣而不做此事

12、由于视力原因，您在挑选及搭配衣服时有多大困难？( )

A.没有困难 B.有一点困难 C.有困难(中等程度) D.的确很困难

E.由于视力原因已不再做此事 F.由于其它原因或没有兴趣而不做此事

13、由于视力原因，您在去别人家作客、参加聚会或者在餐厅就餐时有多大困难? ( )

A.没有困难 B.有一点困难 C.有困难(中等程度) D.的确很困难

E.由于视力原因已不再做此事 F.由于其它原因或没有兴趣而不做此事

14、由于视力原因，您在外出看电影、演出或体育比赛上有多大困难? ( )

A.没有困难 B.有一点困难 C.有困难(中等程度) D.的确很困难

E.由于视力原因已不再做此事 F.由于其它原因或没有兴趣而不做此事

15、您最近有没有骑自行车或驾驶机动车(摩托车，汽车)? ( )

A.有(跳至15c) B.没有

15A、如果没有：您从不骑(驾)车还是您已经停止骑(驾)车了? ( )

A.从不骑(驾)车 (跳至17) B.停止骑(驾)车

15B、如果您停止骑(驾)车，主要是由于您的视力原因还是其它原因? ( )

A.主要是视力原因(跳至17) B.其它原因(跳至17) C.视力及其它原因均有(跳至17)

15C、如果您最近有骑(驾)车，白天在熟悉的地方您在骑(驾)车时有多大困难? ( )

A.没有困难 B.有一点困难 C.有困难(中等程度) D.的确很困难

16、在夜间骑(驾)车，您有多大困难? ( )

A.没有困难 B.有一点困难 C.有困难(中等程度) D.的确很困难

E.由于视力原因已不再做此事 F.由于其它原因或没有兴趣而不做此事

16A、在困难条件下骑(驾)车，例如在差的天气情况，交通高峰时段，高速公路上或交通拥挤

时，您有多大困难? ( )

A.没有困难 B.有一点困难 C.有困难(中等程度) D.的确很困难

E.由于视力原因已不再做此事 F.由于其它原因或没有兴趣而不做此事

第三部分 视力问题的反映

17、由于视力原因，您是否经常不能完成预期的工作目标? ( )

A.一直是这样 B.大多数情况下是这样 C.有时候是这样 D.偶尔是这样 E.没有

18、由于视力原因，您工作或做其它事情的时间是否受限? ( )

A.一直是这样 B.大多数情况下是这样 C.有时候是这样 D.偶尔是这样 E.没有

19、是否经常出现由于眼睛及周围疼痛、不适感而影响或妨碍到做您想要做的事情? ( )

A.一直是这样 B.大多数情况下是这样 C.有时候是这样 D.偶尔是这样 E.没有

20、由于视力问题，我大多数时间等候待在家中 ( )

A.的确是这样 B.大概是这样 C.不确定 D.大概不是这样 E.确实不是这样

2l、由于视力问题，我很多时间感到灰心丧气 ( )

A.的确是这样 B.大概是这样 C.不确定 D.大概不是这样 E.确实不是这样

22、由于视力问题，我很难对我所傲的事情进行控制 ( )

A.的确是这样 B.大概是这样 C.不确定 D.大概不是这样 E.确实不是这样

23、由于视力问题，我不得不更多地依赖他人告诉我很多事情 ( )

A.的确是这样 B.大概是这样 C.不确定 D.大概不是这样 E.确实不是这样

24、由于视力问题，我需要从别人那里得到更多地帮助 ( )

A.的确是这样 B.大概是这样 C.不确定 D.大概不是这样 E.确实不是这样

25、由于视力问题，我担心做出一些令自己或他人尴尬的事情 ( )

A.的确是这样 B.大概是这样 C.不确定 D.大概不是这样 E.确实不是这样

**3.Samples from participants**

**Sample 1**

**
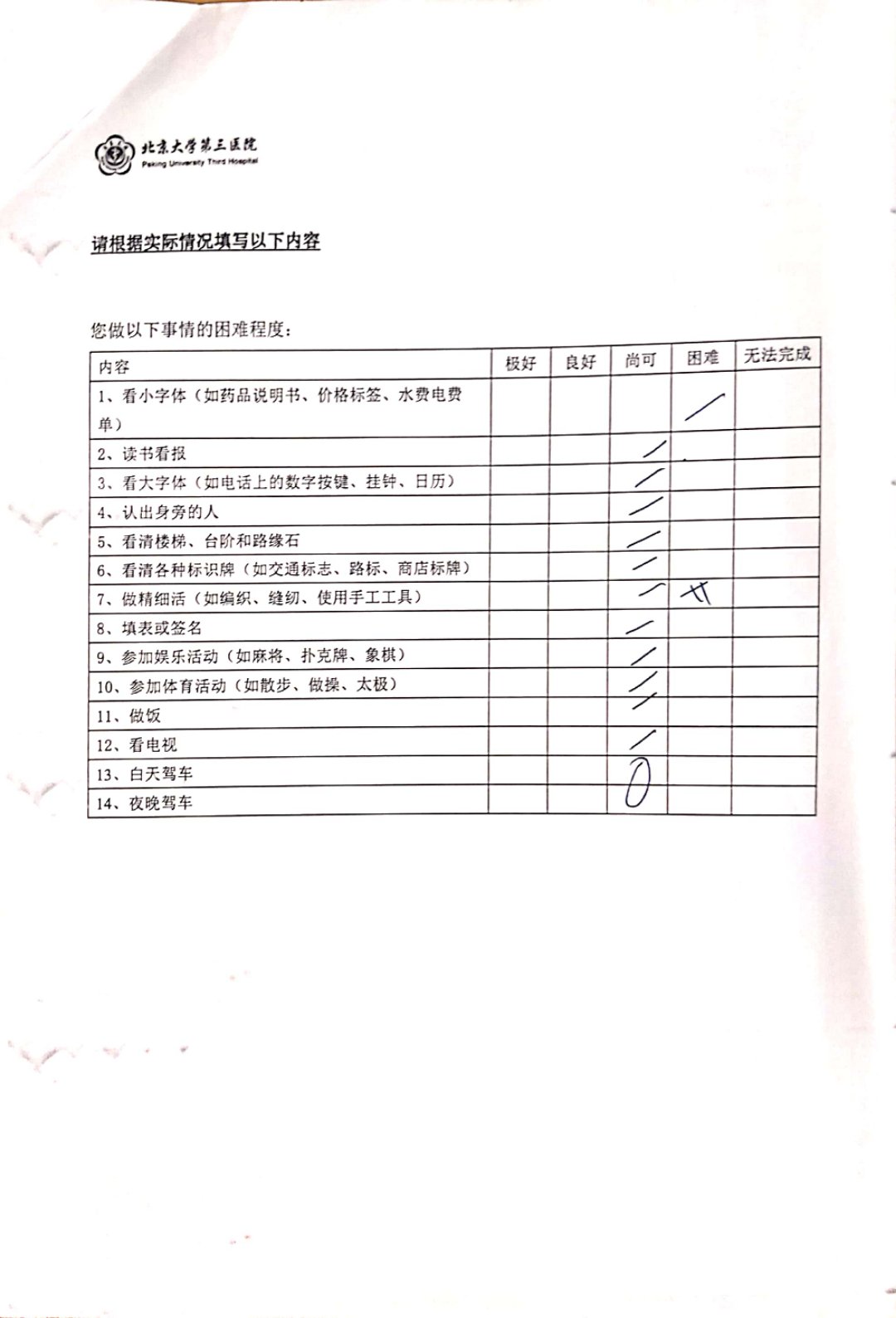
**

**
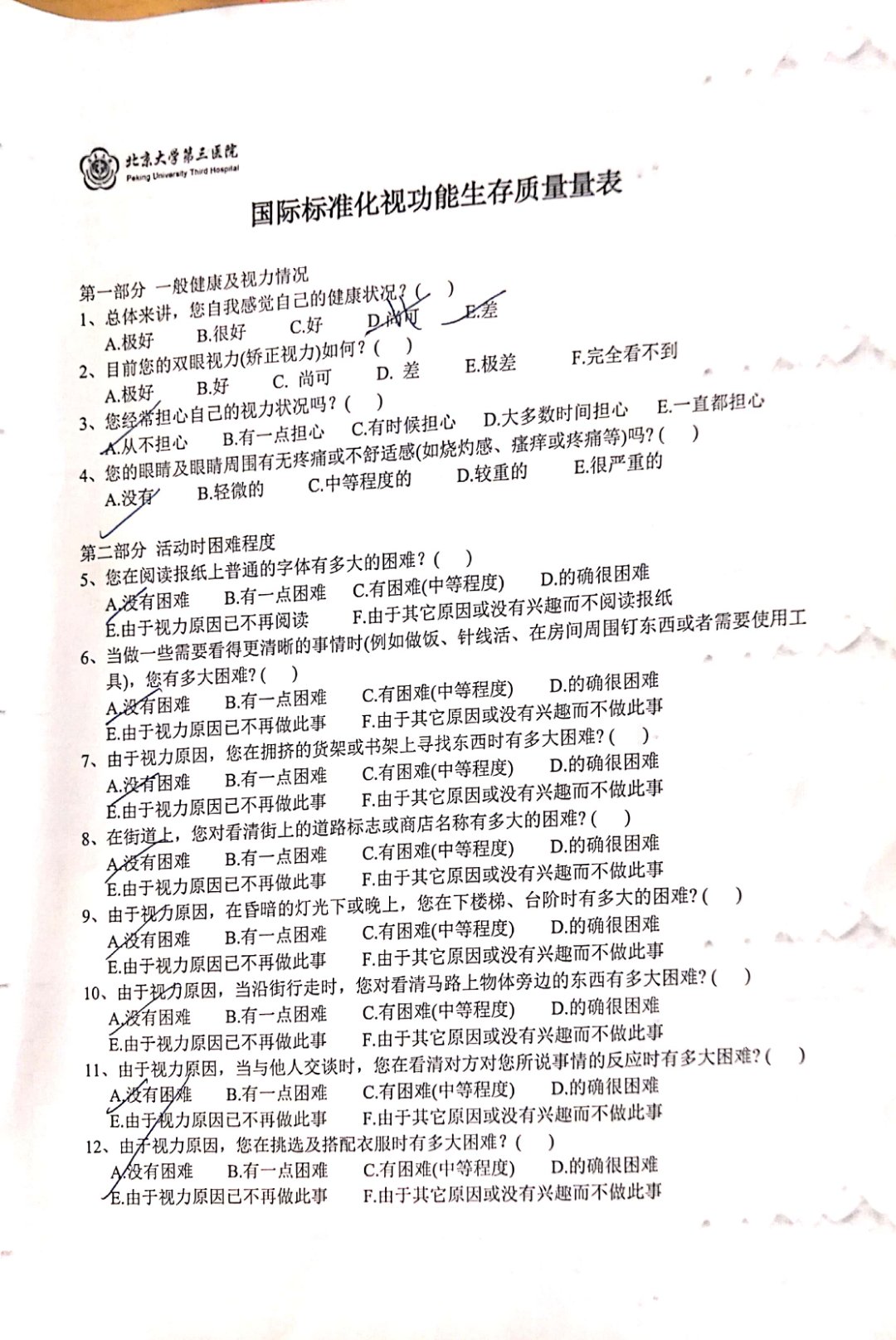
**


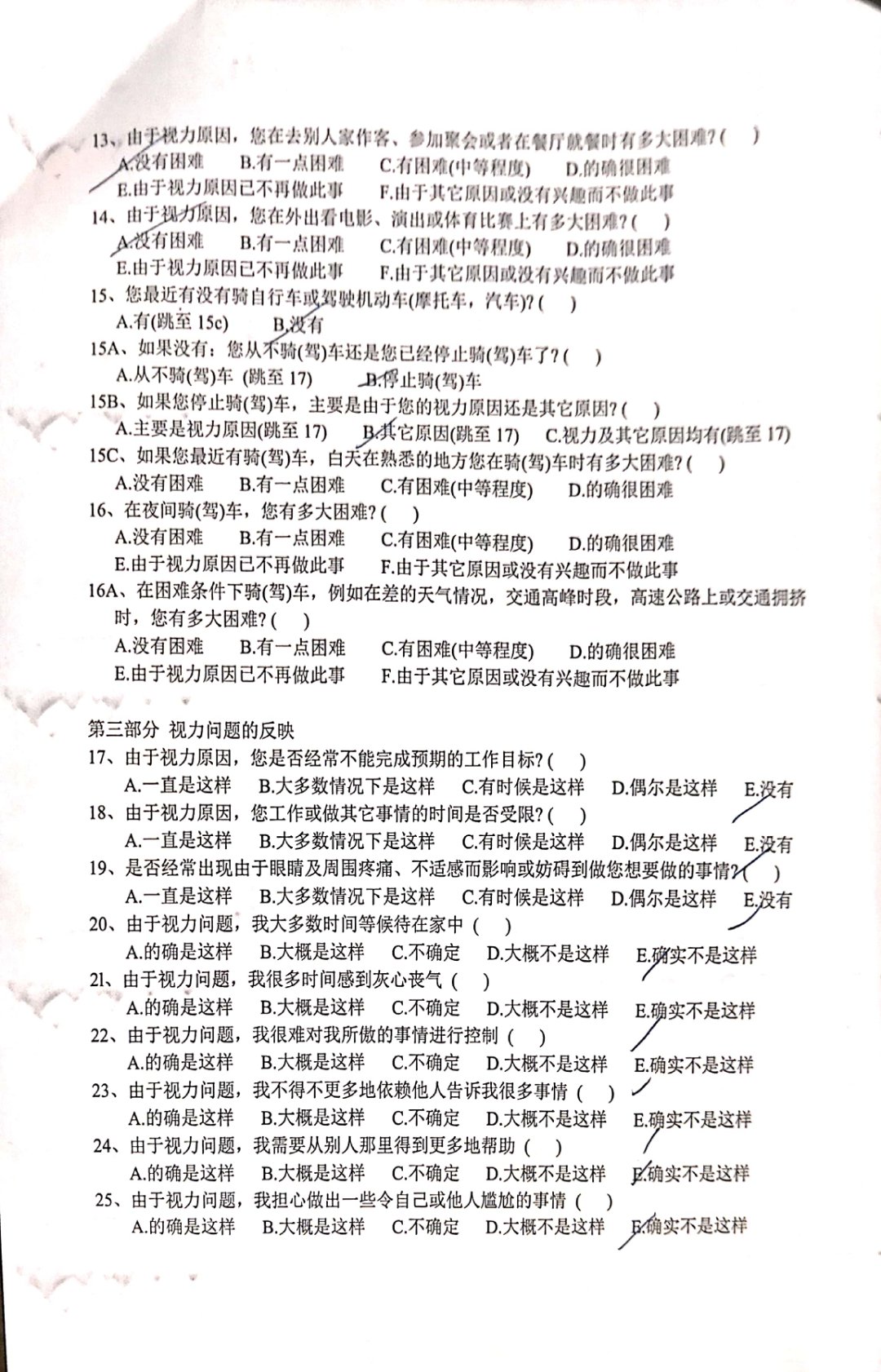


**Sample 2**


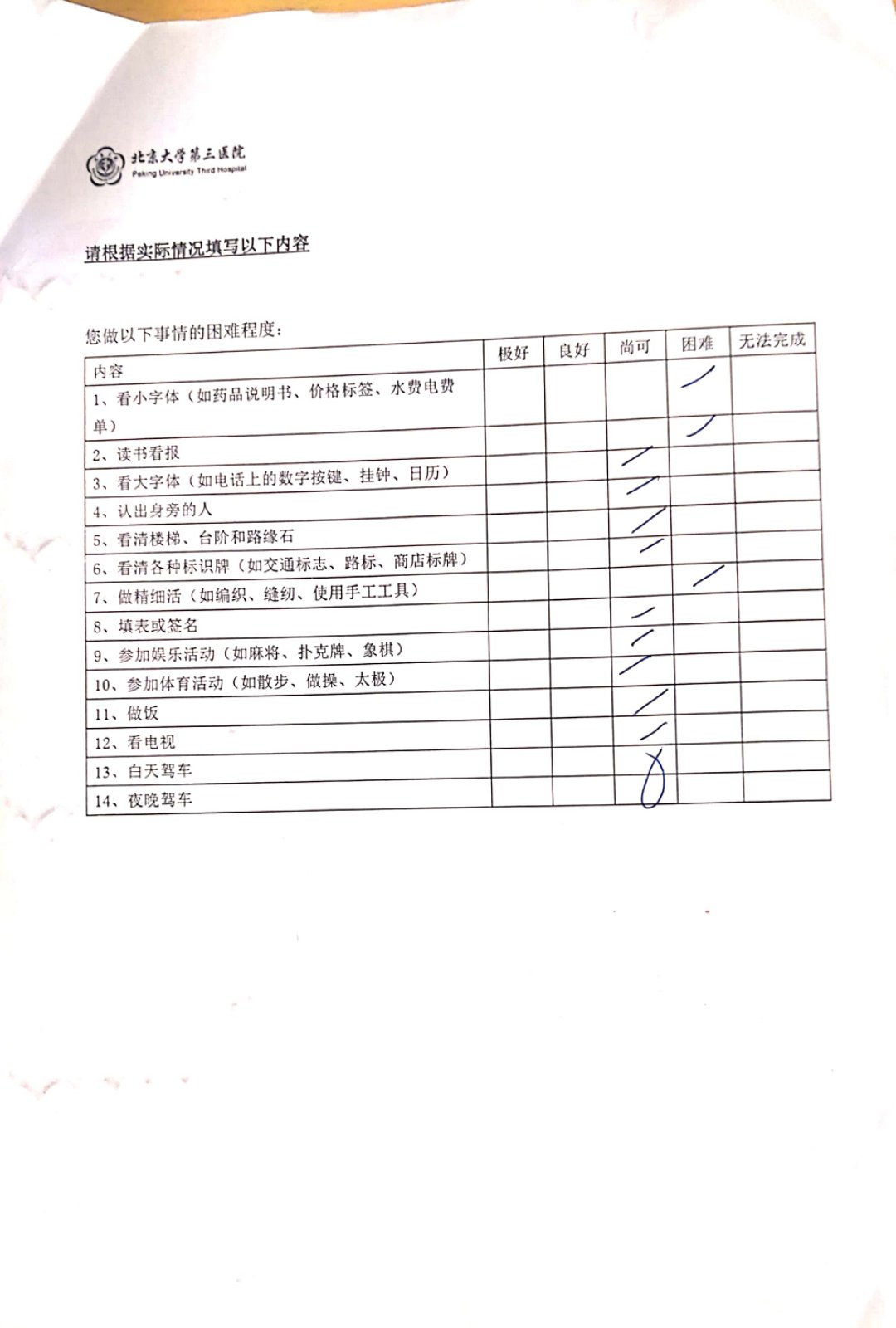


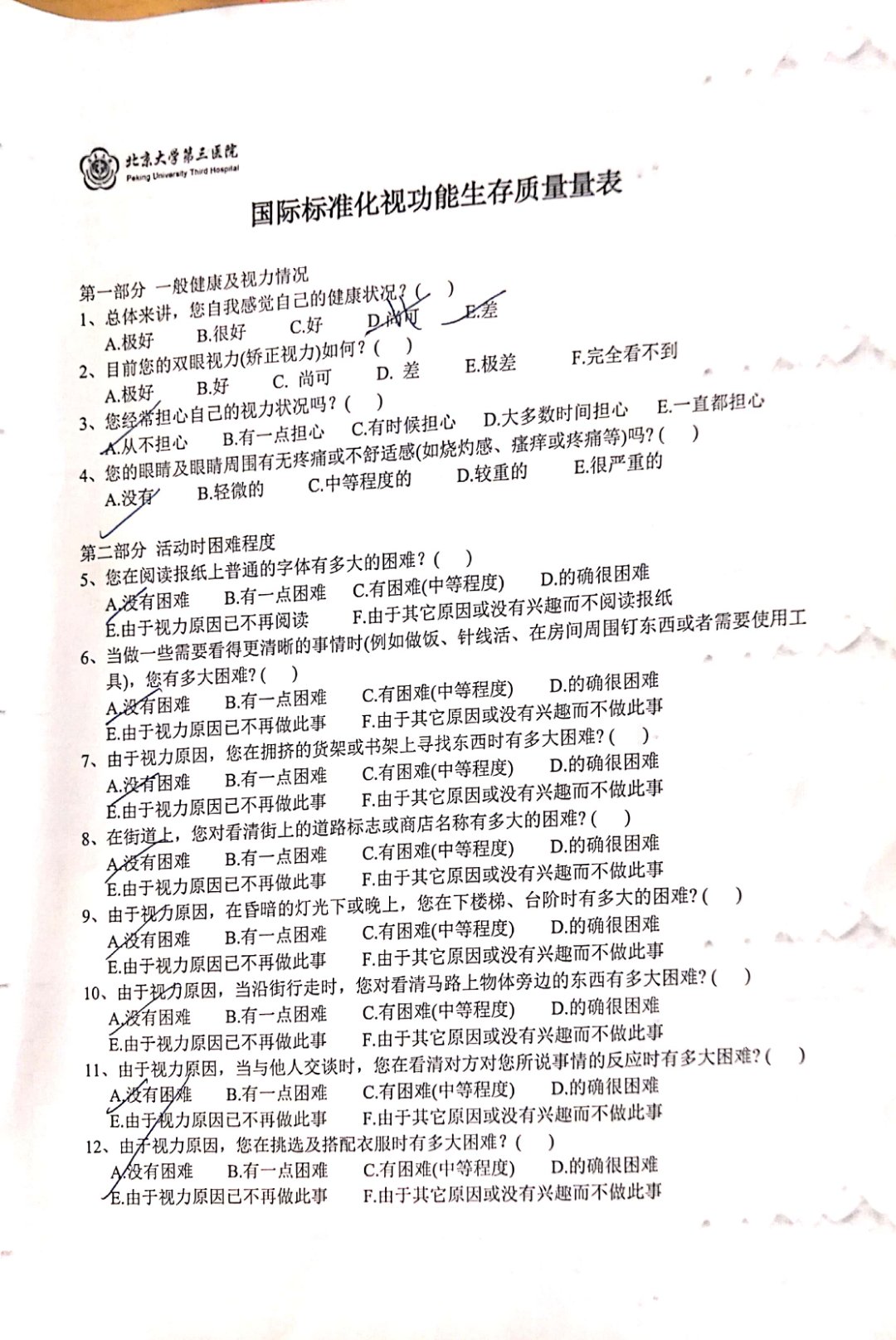


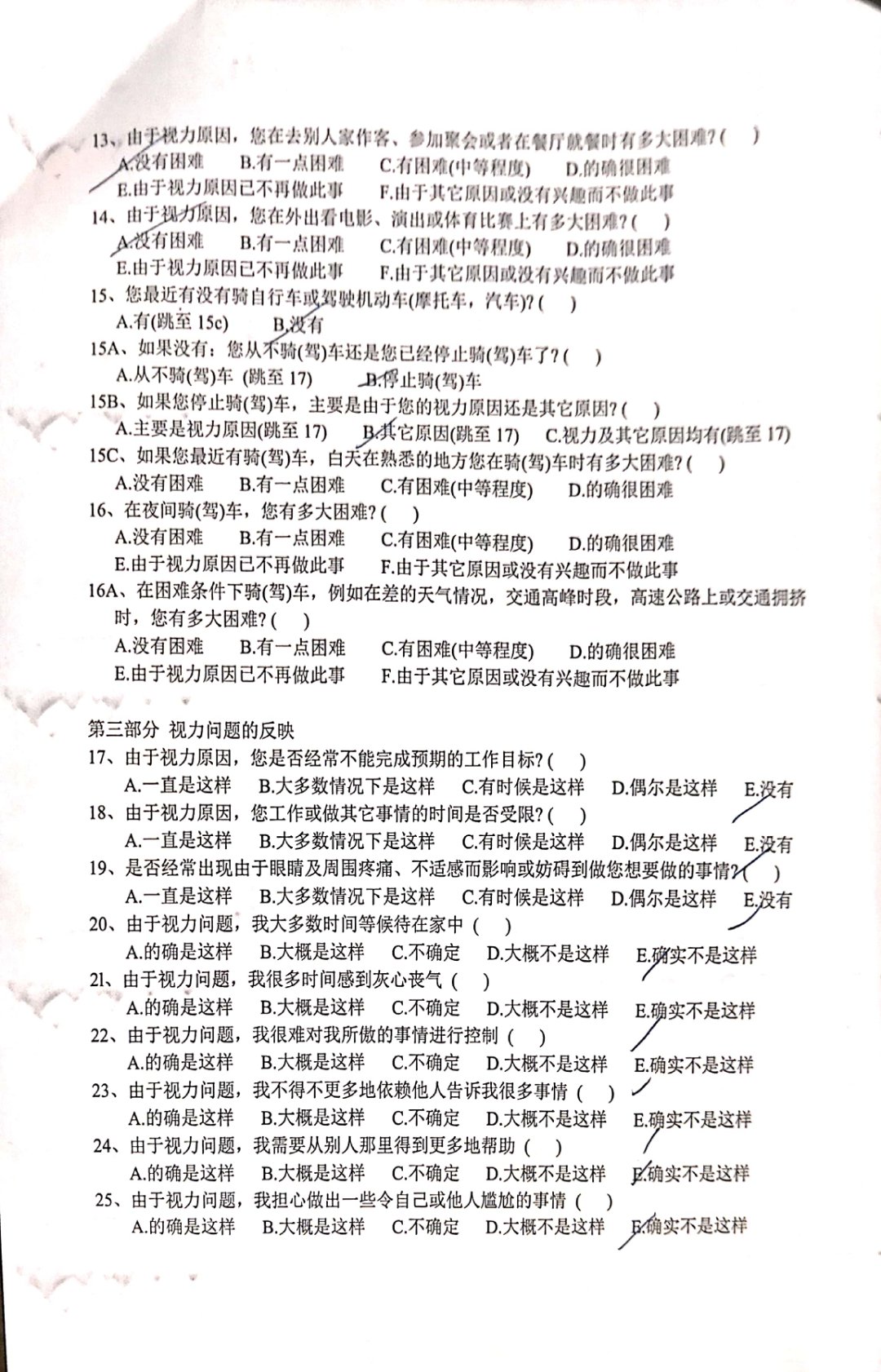

Supplement: Supplementary file 1 — Supplementary material 1 (DOCX 2105 kb) [file 10792_2020_1353_MOESM1_ESM.docx]
