# Supplementary material for: Correlation among Lens Opacities Classification System III grading, the 25-item National Eye Institute Visual Functioning Questionnaire, and Visual Function Index-14 for age-related cataract assessment
Source: Int Ophthalmol. 2020 Apr 5;40(7):1831–9. doi: 10.1007/s10792-020-01353-0 (PMC7308262; doi:10.1007/s10792-020-01353-0)
Supplement: Supplementary file 2 — Supplementary material 2 (DOCX 17 kb) [file 10792_2020_1353_MOESM2_ESM.docx]

**Supplementary Table. Comparison of UDVA (LogMAR) of op-eye, VF-14 and NEI-VFQ-25 between Group C1 and Group C2**

|  | Group C1 | | Group C2 | | P |
| --- | --- | --- | --- | --- | --- |
|  | Mean (SD) | Median | Mean (SD) | Median |  |
| UDVA of op-eye | 0.69 (0.38) | 0.60 | 0.83 (0.38) | 0.82 | <0.001 |
| VF-14 | 49.53 (13.64) | 46.43 | 44.03 (15.76) | 43.75 | <0.001 |
| General Health | 40.99 (18.65) | 50.00 | 38.43 (20.74) | 50.00 | 0.078 |
| General Vision | 47.39 (16.04) | 40.00 | 45.32 (17.72) | 40.00 | 0.056 |
| Ocular Pain | 88.70 (17.72) | 100.00 | 81.16 (22.82) | 87.50 | <0.001 |
| Near Activities | 71.63 (24.08) | 83.33 | 65.34 (24.84) | 66.67 | <0.001 |
| Distance Activities | 82.37 (20.96) | 91.67 | 73.22 (25.07) | 75.00 | <0.001 |
| Social Functioning | 91.14 (18.77) | 100.00 | 82.44 (24.43) | 100.00 | <0.001 |
| Mental Health | 84.23 (21.08) | 93.75 | 73.93 (26.93) | 87.50 | <0.001 |
| Role Difficulties | 78.87 (26.58) | 100.00 | 64.75 (30.11) | 50.00 | <0.001 |
| Dependency | 85.86 (24.07) | 100.00 | 76.80 (28.76) | 100.00 | <0.001 |
| Driving | 81.17 (31.87) | 100.00 | 65.57 (36.44) | 75.00 | <0.001 |
| Color Vision | 91.21 (22.20) | 100.00 | 82.69 (26.90) | 100.00 | <0.001 |
| Peripheral Vision | 84.01 (23.64) | 100.00 | 75.48 (26.71) | 75.00 | <0.001 |
| Total Score of NEI-VFQ-25 | 80.71 (15.78) | 86.29 | 71.96 (20.47) | 76.71 | <0.001 |

SD, standard deviation; UDVA, uncorrected distance visual acuity; Op-eye, operative eye; VF-14, Visual Function Index-14; NEI-VFQ-25, 25-item National Eye Institute Visual Functioning Questionnaire
